# Data-Driven Analysis of Nonlinear Heterogeneous Reactions through Sparse Modeling and Bayesian Statistical Approaches

**DOI:** 10.3390/e23070824

**Published:** 2021-06-28

**Authors:** Masaki Ito, Tatsu Kuwatani, Ryosuke Oyanagi, Toshiaki Omori

**Affiliations:** 1 Graduate School of Engineering, Kobe University, 1-1 Rokkodai-cho, Nada-ku, Kobe 657-8501, Japan; en818.businhete2021@gmail.com; 2 Research Institute for Marine Geodynamics, Japan Agency for Marine-Earth Science and Technology (JAMSTEC), 2-15 Natsushima-cho, Kanagawa, Yokosuka 237-0061, Japan; kuwatani@jamstec.go.jp (T.K.); oyanagir@kokushikan.ac.jp (R.O.); 3 School of Science and Engineering, Kokushikan University, 4-28-1 Setagaya, Setagaya-ku, Tokyo 154-8515, Japan; 4 Center for Mathematical and Data Sciences, Kobe University, 1-1 Rokkodai-cho, Nada-ku, Kobe 657-8501, Japan

**Keywords:** sparse modeling, sequential Monte Carlo method, time series data analysis, heterogeneous reactions

## Abstract

Heterogeneous reactions are chemical reactions that occur at the interfaces of multiple phases, and often show a nonlinear dynamical behavior due to the effect of the time-variant surface area with complex reaction mechanisms. It is important to specify the kinetics of heterogeneous reactions in order to elucidate the microscopic elementary processes and predict the macroscopic future evolution of the system. In this study, we propose a data-driven method based on a sparse modeling algorithm and sequential Monte Carlo algorithm for simultaneously extracting substantial reaction terms and surface models from a number of candidates by using partial observation data. We introduce a sparse modeling approach with non-uniform sparsity levels in order to accurately estimate rate constants, and the sequential Monte Carlo algorithm is employed to estimate time courses of multi-dimensional hidden variables. The results estimated using the proposed method show that the rate constants of dissolution and precipitation reactions that are typical examples of surface heterogeneous reactions, necessary surface models, and reaction terms underlying observable data were successfully estimated from only observable temporal changes in the concentration of the dissolved intermediate products.

## 1. Introduction

Specifying the underlying dynamical mechanism from observed time-series data sets is ubiquitously important in natural sciences [1]. Generally, the governing equations include many candidate nonlinear terms for describing complex dynamical behaviors, and the observations of the actual problems are always limited [1,2]. Therefore, in order to understand the microscopic elementary processes and predict the future macroscopic behaviors of a complex system, it is necessary to develop a data-driven method that can extract substantial elements from a number of candidates by using partially observable time-series data.

Heterogeneous reactions are chemical reactions that occur at the interface of multiple phases, and they are essential to understand the complex behavior of substances in natural systems [3]. Their dynamics have intrinsic nonlinearity caused by the effect of inevitable changes in the surface area among different phases [2]. In earth science, it is important to understand heterogeneous reactions in order to clarify the dynamics of rock formations near the surface of the earth. However, in heterogeneous reactions, there are many kinds of reaction terms with different surface models and orders of reactions [2]. Therefore, it is difficult to extract important reactions from many candidates in order to estimate the underlying dynamics of heterogeneous reactions from the observable data.

Bayesian statistical approaches were proposed to estimate heterogeneous reactions from observable data. A Bayesian inversion analysis method with sequential Monte Carlo and expectation–maximization (EM) algorithms was proposed to simultaneously estimate the kinetic rate constants and time series of multi-dimensional hidden variables for heterogeneous reactions [4]. Moreover, a Bayesian approach, using Markov chain Monte Carlo and widely applicable information criteria, was proposed to estimate heterogeneous reaction pathways using spatial data [5]. In these previous Bayesian methods [4,5], the types of reaction terms are assumed to be known. However, it is important to establish a method for estimating reaction constants while assuming that the types of reaction terms are unknown since there exist many kinds of candidates in reaction terms in heterogeneous reactions.

Recently, sparse modeling approaches have attracted much attention owing to their ability to extract important factors from observable data [6,7]. The sparse modeling approach can extract essential elements from candidate factors that reconstruct observed data by assuming that important elements within the entire candidate elements are sparse. Sparse modeling is widely employed in various fields of natural sciences, such as physics [8,9], brain science [10,11,12], astronomy [13] and earth sciences [14]. In particular, applying the sparse modeling approach to heterogeneous reactions is an important subject since sparse modeling may realize the extraction of only necessary terms from many candidates of reaction terms.

This paper proposes a data-driven method to simultaneously estimate rate constants and hidden variables from nonlinear dynamics of heterogeneous reactions of fluid–rock interaction by adapting sparse modeling and sequential Monte Carlo methods. First, we establish a state-space model for nonlinear dynamics for heterogeneous reactions, including multiple candidates of the reaction terms. Next, we derive the sequential Monte Carlo method for partial observation problems in heterogeneous reactions with many candidate terms. Furthermore, we adapt the sparse modeling approach to estimation problem of dynamical system in order to estimate necessary rate constants from many candidates. We employ a sparse modeling approach with non-uniform sparsity levels and make the sparse modeling algorithm more robust in order to improve the estimation accuracy for rate constants. The estimated results show that the proposed method can extract necessary reaction terms from many candidates more accurately, compared with naïve estimation methods.

This paper is organized as follows. In Section 2, we propose a sparse modeling method for extracting heterogeneous reactions. First, we describe a general form of differential equations of heterogeneous reactions. Next, we derive a nonlinear state-space model for the heterogeneous reactions, and then we adapt a sparse modeling approach to probabilistic frameworks described by the state-space model of heterogeneous reactions. In Section 3, we verify the effectiveness of the proposed method, using simulated data obtained from a dynamical model of heterogeneous reaction while assuming many candidates of nonlinear terms. Concluding remarks are given in Section 4.

## 2. Method

In this section, we propose a sparse modeling method for extracting heterogeneous reactions. First, we introduce heterogeneous reactions, which depend on surface-area reactions between the liquid phase and the solid phase, and formulate a nonlinear state-space model of heterogeneous reactions with many candidate terms. Next, we employ the sequential Monte Carlo method in order to estimate the hidden variables of heterogeneous reactions from observable data. Finally, we adopt a sparse modeling approach to the framework of the sequential Monte Carlo method in order to extract important heterogeneous reactions from many candidates.

### 2.1. Nonlinear Dynamics of Heterogeneous Reactions of Fluid–Rock Interaction

In this study, we consider a general setting of heterogeneous reactions in fluid–rock interactions in which minerals (solid phase) dissolve and precipitate within a liquid phase. The fluid–rock interaction is one of the most important geoscientific processes in the earth. For example, serpentinization, a collection of heterogeneous reactions to generate serpentinite from peridotite and water, in the oceanic crust is known to be a key storage process of water brought into the earth’s deep region. In addition, it changes the physical properties of the earth’s interior significantly, and affects the occurrence of earthquakes in the subduction zone [15]. It is important to specify the kinetics of heterogeneous reactions in order to understand the detailed processes of rock formation and predict the evolution of the earth system. However, estimating the heterogeneous reactions is difficult since observation data are limited, and there are many candidates for reaction types [16,17,18,19]. In addition, dominant reaction terms generally vary according to external conditions, such as temperature and pressure, hence it is very difficult to presumably predict which terms are active or inactive. Therefore, it is imperative to establish a method for estimating the reactions based on data-driven analysis using the data sets that are obtained from laboratory experiments and natural samples [20].

**Figure 1:**
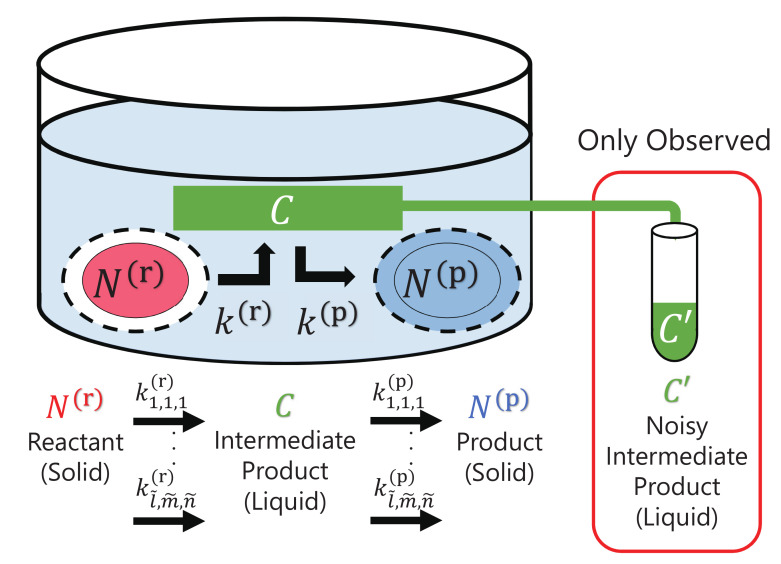
Schematic diagram of surface heterogeneous reactions consisting of a reactant $N^{\left(r\right)}$, intermediate product *C* and product $N^{\left(p\right)}$. The solid reactant dissolves into a liquid intermediate product and it precipitates into a solid product, and there are many candidate reaction terms for the surface heterogeneous reactions. These reactions depend on rate constants $k_{l,m,n}^{\left(r\right)}$ and $k_{l,m,n}^{\left(p\right)}$ for different surface-area reactions $S_{l}^{\left(r\right)}(N^{\left(r\right)})$ and $S_{l}^{\left(p\right)}(N^{\left(p\right)})$, and different orders of reaction terms *m* and *n*.

Here, we consider surface heterogeneous reactions in a hydrothermal laboratory experiment as an analogue of fluid–rock interactions in natural systems, as shown in Figure 1. In the reactions, a solid reactant $N^{\left(r\right)}$ dissolves into a liquid to form an intermediate product *C*, and the intermediate product *C* precipitates to form a solid product $N^{\left(p\right)}$.

Such dissolution and precipitation via an intermediate product within fluid is considered to be the most fundamental process, which describes the essence of heterogeneous reactions in fluid–rock interactions. Since there are many kinds of heterogeneous reactions that are linear or nonlinear related to surface-area reactions and reaction terms, and we cannot presumably assume effective specific reaction terms, we consider the amounts of reactant $N^{\left(r\right)}$, and those of product $N^{\left(p\right)}$ obey the following general differential equations with a number of candidate reaction terms [2]:

(1) $$\frac{dN^{\left(r\right)}}{dt^{\prime }}=\sum_{l=1}^{\tilde{l}}\sum_{m=1}^{\tilde{m}}\sum_{n=1}^{\tilde{n}}k_{l,m,n}^{\left(r\right)}S_{l}^{\left(r\right)}\left(N^{\left(r\right)}\right){\left\{C^{m}-{\left(C_{eq}^{\left(r\right)}\right)}^{m}\right\}}^{n}$$

(2) $$\frac{dN^{\left(p\right)}}{dt^{\prime }}=\sum_{l=1}^{\tilde{l}}\sum_{m=1}^{\tilde{m}}\sum_{n=1}^{\tilde{n}}k_{l,m,n}^{\left(p\right)}S_{l}^{\left(p\right)}\left(N^{\left(p\right)}\right){\left\{C^{m}-{\left(C_{eq}^{\left(p\right)}\right)}^{m}\right\}}^{n}$$

(3) $$\frac{dN^{\left(r\right)}}{dt^{\prime }}+\frac{dC}{dt^{\prime }}+\frac{dN^{\left(p\right)}}{dt^{\prime }}=0$$

where $t^{\prime }$ denotes time. Here, $k_{l,m,n}^{\left(r\right)}$ and $k_{l,m,n}^{\left(p\right)}$ are the rate constants governing the dynamics of heterogeneous reactions. $S_{l}^{\left(r\right)}\left(N\right)$ and $S_{l}^{\left(p\right)}\left(N\right)$ indicate the surface areas of the reactant and product, respectively. $l\;(l=1,2,\cdots ,\tilde{l})$ is a type of surface area model. $m\;(m=1,2,\cdots ,\tilde{m})$ and $n\;(n=1,2,\cdots ,\tilde{n})$ are indices for the orders of an intermediate product *C* and factor $\left\{C^{m}-{\left(C_{eq}\right)}^{m}\right\}$ of the reaction terms, respectively. Here, $\tilde{l}$, $\tilde{m}$ and $\tilde{n}$ are the total numbers of candidate surface models and candidate orders of the two factors. Note that Equations (1) and (2) contain $\tilde{l}\times \tilde{m}\times \tilde{n}$ kinds of candidate reaction terms for each equation, and essential reaction terms are extracted from the candidates, using the framework of sparse modeling as described below.

**Figure 2:**
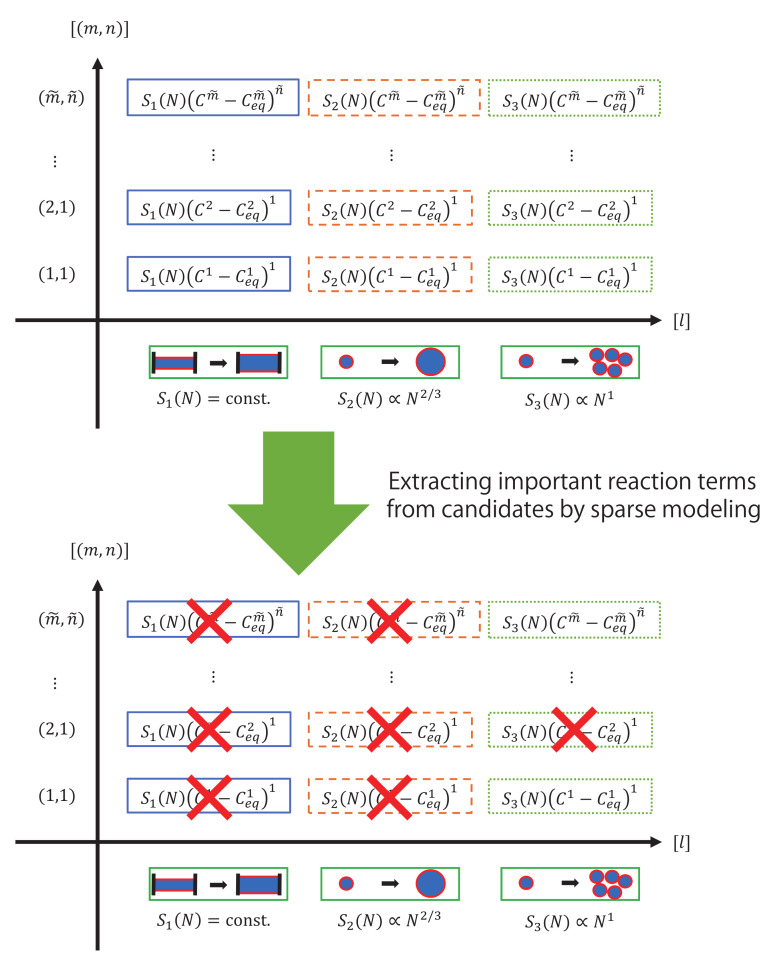
Extraction of important reaction terms from many reaction candidates. (Top) reaction candidates in heterogeneous reactions. The horizontal line shows candidates for surface models, whereas the vertical line shows candidates for the orders of reaction terms. We consider M×N×L candidate terms with different surface models ($S_{1}\left(N\right)=\mathrm{const}.$, $S_{2}\left(N\right)\propto N^{2/3}$, and $S_{3}\left(N\right)\propto N$) and different orders *m* and *n* in factors of reaction terms ${\left\{C^{m}-{\left(C_{eq}\right)}^{m}\right\}}^{n}$. (Bottom) example of extraction of important reaction terms.

The dynamics of heterogeneous reactions shown in Equations (1)–(3) depend on the rate constants $k_{l,m,n}^{\left(r\right)}$ and $k_{l,m,n}^{\left(p\right)}$. The constants indicate how fast the reactants $N^{\left(r\right)}$ dissolve and the products $N^{\left(p\right)}$ precipitate. Therefore, rate constants $k_{l,m,n}^{\left(r\right)}$ and $k_{l,m,n}^{\left(p\right)}$ are important factors to determine what kind of mineral dissolves into a liquid intermediate product and precipitates into a solid product. Figure 2 shows our problem setting for extracting important reactions from many reaction candidates. In Figure 2, surface area is simply expressed as $S_{l}\left(N\right)$ rather than $S_{l}^{\left(r\right)}\left(N\right)$ and $S_{l}^{\left(p\right)}\left(N\right)$. $S_{l}\left(N\right)$ indicates the surface area between two solid and liquid phases, and there are many types of surface-area reactions that highly influence heterogeneous surface reactions. Generally, a surface area $S_{l}\left(N\right)$ is expressed as follows:

(4) $$S_{l}\left(N\right)\propto N^{\alpha }$$

where α is the order of the surface-area reaction. Note that the surface area $S_{l}\left(N\right)$ depends on the number *N* of moles of the solid phase. As shown in Equation (4), $S_{l}\left(N\right)$ is proportional to the α-th power of *N*. It is necessary to consider the kind of surface-area reaction that occurs since heterogeneous reactions change at the interface between the solid and liquid phases.

In this study, we consider three typical surface models (Figure 2, horizontal line) as follows:

(5) $$\begin{array}{ccc} S_{1}\left(N\right) & = & \mathrm{const}. \end{array}$$

(6) $$\begin{array}{ccc} S_{2}\left(N\right) & \propto & N^{\frac{2}{3}} \end{array}$$

(7) $$\begin{array}{ccc} S_{3}\left(N\right) & \propto & N \end{array}$$

In Figure 2, the blue area indicates the solid phase, whereas the red lines represent the surface between the solid phase (blue area) and liquid phase (white area). For α=0, the surface area does not change with an increase or decrease in the amount of the solid phase. For α=2/3, the geometrical shape of the solid phase does not change with an increase or decrease in the amount of the solid phase. For α=1, the bulk reaction occurs, and the surface area changes in proportion to the amount of solid phase.

As shown in the vertical line of Figure 2, there are candidates for indices of nonlinearity *m* and *n* in the reaction terms, where $m\;(m=1,2,\cdots ,\tilde{m})$ is a multiplier of the intermediate product *C*. $n\;(n=1,2,\cdots ,\tilde{n})$ is a multiplier of the reaction terms $\left\{C^{m}-{\left(C_{eq}\right)}^{m}\right\}$. Both $C^{m}$ and ${\left\{C^{m}-{\left(C_{eq}\right)}^{m}\right\}}^{n}$ are important for the dynamics of heterogeneous reactions.

**Figure 3:**
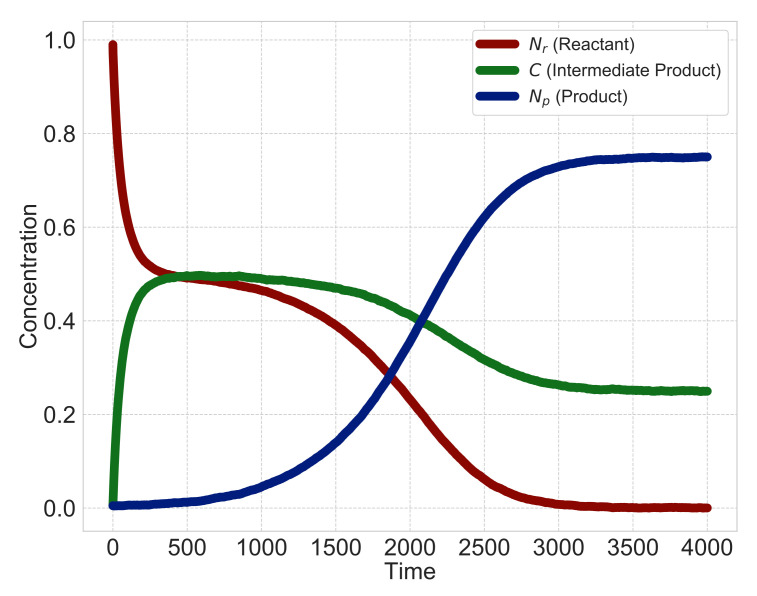
Typical behavior of heterogeneous reactions. At t=0, a solid reactant $N^{\left(r\right)}$ only exists and dissolves into a liquid intermediate product *C* as time passes. When the intermediate product *C* dissolves sufficiently, it precipitates into a solid product $N^{\left(p\right)}$. With more time, the solid reactant $N^{\left(r\right)}$ almost disappears, and the intermediate product *C* and the solid product $N^{\left(p\right)}$ become constant.

Figure 3 shows a typical time course of the heterogeneous reaction when the type of surface-area model is l=3 and the multipliers m=1, n=1 in Equations (1) and (2). We simulate differential equations, Equations (1)–(3), with discretized time step *t* and set the initial values $N^{\left(r\right)}\left(0\right)=0.99$, C(0)=0.005 and $N^{\left(p\right)}\left(0\right)=0.005$. As shown in Figure 3, when the time is zero, the solid reactant $N^{\left(r\right)}$ exists and dissolves into a liquid intermediate product *C* with time. When the intermediate product *C* dissolves sufficiently, it precipitates into a solid product $N^{\left(p\right)}$. After that, the solid reactant $N^{(}r)$ almost disappears, and the intermediate product *C* and solid product $N^{\left(p\right)}$ become constant.

### 2.2. Estimating Heterogeneous Reaction Dynamics Based on Sequential Monte Carlo Method

In order to estimate the rate constants $k=\left\{k_{l,m,n}^{\left(r\right)}k_{l,m,n}^{\left(p\right)}\right\}$, it is necessary to estimate the solid reactant $N^{\left(r\right)}$, liquid intermediate product *C* and solid product $N^{\left(p\right)}$. We consider how the reactants $N^{\left(r\right)}$, intermediate products *C* and products $N^{\left(p\right)}$ change over time.

**Figure 4:**
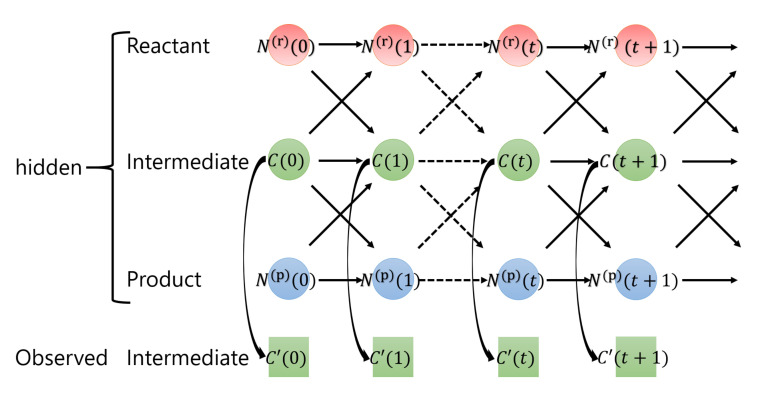
Graphical model of a nonlinear state-space model for heterogeneous reactions. The reactant $N^{\left(r\right)}\left(t+1\right)$ at time t+1 depends on the reactant $N^{\left(r\right)}\left(t\right)$ and the intermediate product C(t) at time *t*. In addition, the reactant $N^{\left(r\right)}\left(t\right)$, intermediate product C(t) and product $N^{\left(p\right)}\left(t\right)$ affect the intermediate product C(t+1) and product $N^{\left(p\right)}\left(t+1\right)$. Moreover, since only the intermediate product C(t) is observable, an observable variable $C^{\prime }\left(t\right)$ is assumed to be generated from intermediate product C(t) at each time.

Figure 4 shows the graphical model of surface heterogeneous reactions governed by Equations (1)–(3). Here, we consider discretized time steps for the states of surface heterogeneous reactions: the reactant $N^{\left(r\right)}\left(t+1\right)$ at time step t+1 depends on the reactant $N^{\left(r\right)}\left(t\right)$ and the intermediate product C(t) at the preceding time step *t*. Additionally, the reactant $N^{\left(r\right)}\left(t\right)$, the intermediate product C(t) and the product $N^{\left(p\right)}\left(t\right)$ affect the intermediate product C(t+1) and the product $N^{\left(p\right)}\left(t+1\right)$. Moreover, since the intermediate product is assumed to be observable, the observed intermediate product $C^{\prime }\left(t\right)$ has a relationship with the intermediate product C(t) at the respective time.

From Figure 4, we propose a nonlinear state-space model for surface heterogeneous reactions. The state space model is a probabilistic model that describes the time evolution and observation process of the dynamical system, and was used in the time series analysis for estimating and predicting hidden variables in dynamical system from observable time-series data [21,22]. The state-space model consists of observation and system models [23]. The observation model represents the relationship between hidden and observation variables, whereas the system model represents the relationship between hidden variables at two adjacent times. In this study, the solid reactant $N^{\left(r\right)}$, liquid intermediate product *C* and solid product $N^{\left(p\right)}$ are hidden variables, and the observed liquid intermediate product $C^{\prime }$ is an observation variable.

First, we formulate the observation model by assuming that the observed liquid intermediate product $C^{\prime }\left(t\right)$ is obtained as a sum of the true liquid intermediate product C(t) and an additive noise as follows:

(8) $$\begin{array}{c} \begin{array}{c} C^{\prime }\left(t\right)=C\left(t\right)+\xi^{C}\left(t\right) \end{array} \end{array}$$

where $\xi^{C}\left(t\right)$ denotes an observation noise. Assuming that the observation noise obeys the Gaussian distribution, the observation model is expressed by a probabilistic model as follows:

(9) $$\begin{array}{c} \begin{array}{c} p\left(C^{\prime }\left(t\right)|C\left(t\right)\right)=\frac{1}{\sqrt{2\pi \sigma_{y}^{2}}}\exp\left(-\frac{{\left(C^{\prime }\left(t\right)-C\left(t\right)\right)}^{2}}{2\sigma_{y}^{2}}\right) \end{array} \end{array}$$

where $\sigma_{y}$ shows a standard deviation of the observation noise.

Next, we derive the system model that expresses the relationship among hidden variables. By discretizing Equations (1)–(3) at the time interval $\Delta_{t}$, we obtain the following difference equation for each time step *t*:

(10) $$\frac{N^{\left(r\right)}\left(t+1\right)-N^{\left(r\right)}\left(t\right)}{\Delta_{t}}=\sum_{l=1}^{\tilde{l}}\sum_{m=1}^{\tilde{m}}\sum_{n=1}^{\tilde{n}}k_{l,m,n}^{\left(r\right)}S_{l}^{\left(r\right)}\left(N^{\left(r\right)}\left(t\right)\right){\left\{C^{m}\left(t\right)-{\left(C_{eq}^{\left(r\right)}\right)}^{m}\right\}}^{n}$$

(11) $$\frac{N^{\left(p\right)}\left(t+1\right)-N^{\left(p\right)}\left(t\right)}{\Delta_{t}}=\sum_{l=1}^{\tilde{l}}\sum_{m=1}^{\tilde{m}}\sum_{n=1}^{\tilde{n}}k_{l,m,n}^{\left(p\right)}S_{l}^{\left(p\right)}\left(N^{\left(p\right)}\left(t\right)\right){\left\{C^{m}\left(t\right)-{\left(C_{eq}^{\left(p\right)}\right)}^{m}\right\}}^{n}$$

(12) $$\frac{N^{\left(r\right)}\left(t+1\right)-N^{\left(r\right)}\left(t\right)}{\Delta_{t}}+\frac{C(t+1)-C(t)}{\Delta_{t}}+\frac{N^{\left(p\right)}\left(t+1\right)-N^{\left(p\right)}\left(t\right)}{\Delta_{t}}=0$$

Furthermore, by assuming a system noise, we obtain the following equations:

(13) $$N^{\left(r\right)}\left(t+1\right)=N^{\left(r\right)}\left(t\right)+\Delta_{t}\sum_{l=1}^{\tilde{l}}\sum_{m=1}^{\tilde{m}}\sum_{n=1}^{\tilde{n}}k_{l,m,n}^{\left(r\right)}S_{l}^{\left(r\right)}\left(N^{\left(r\right)}\left(t\right)\right){\left\{C^{m}\left(t\right)-{\left(C_{eq}^{\left(r\right)}\right)}^{m}\right\}}^{n}+\sqrt{\Delta_{t}}\xi^{\left(r\right)}\left(t\right)$$

(14) $$N^{\left(p\right)}p)\left(t+1\right)=N^{\left(p\right)}\left(t\right)+\Delta_{t}\sum_{l=1}^{\tilde{l}}\sum_{m=1}^{\tilde{m}}\sum_{n=1}^{\tilde{n}}k_{l,m,n}^{\left(p\right)}S_{l}^{\left(p\right)}\left(N^{\left(p\right)}\left(t\right)\right){\left\{C^{m}\left(t\right)-{\left(C_{eq}^{\left(p\right)}\right)}^{m}\right\}}^{n}+\sqrt{\Delta_{t}}\xi^{\left(p\right)}\left(t\right)$$

(15) $$N^{\left(r\right)}\left(t+1\right)-N^{\left(r\right)}\left(t\right)+C\left(t+1\right)-C\left(t\right)+N^{\left(p\right)}\left(t+1\right)-N^{\left(p\right)}\left(t\right)=0$$

where $\xi^{\left(r\right)}\left(t\right)$ and $\xi^{\left(p\right)}\left(t\right)$ denote the system noise obeying the white Gaussian noise: for time steps *t* and *s*, $\langle \xi^{\left(r\right)}\left(t\right)\rangle =\langle \xi^{\left(p\right)}\left(t\right)\rangle =\langle \xi^{\left(r\right)}\left(t\right)\xi^{\left(p\right)}\left(s\right)\rangle =0$, $\langle \xi^{\left(r\right)}\left(t\right)\xi^{\left(r\right)}\left(s\right)\rangle =\sigma_{r}^{2}\delta_{t,s}$, and $\langle \xi^{\left(p\right)}\left(t\right)\xi^{\left(p\right)}\left(s\right)\rangle =\sigma_{p}^{2}\delta_{t,s}$. Here, $\delta_{t,s}$ is a Kronecker delta; $\delta_{t,s}=1$ for t=s and $\delta_{t,s}=0$ for t≠s. $\sigma_{r}$ and $\sigma_{p}$ express the standard deviations of the system noise. Since Equations (13)–(15) have additive Gaussian noise, a probabilistic model of system model for heterogeneous reactions is derived as follows:

(16) $$\begin{array}{cc} \; & p\left(N^{\left(r\right)}\left(t+1\right)|N^{\left(r\right)}\left(t\right),C\left(t\right),N^{\left(p\right)}\left(t\right)\right)=\\ \; & \frac{1}{\sqrt{2\pi {\tilde{\sigma }}_{r}^{2}}}\exp\left(-\frac{{\left(N^{\left(r\right)}\left(t+1\right)-N^{\left(r\right)}\left(t\right)-\Delta_{t}\sum_{l,m,n}k_{l,m,n}^{\left(r\right)}S_{l}^{\left(r\right)}\left(N^{\left(r\right)}\left(t\right)\right){\left\{C^{m}\left(t\right)-{\left(C_{eq}^{\left(r\right)}\right)}^{m}\right\}}^{n}\right)}^{2}}{2{\tilde{\sigma }}_{r}^{2}}\right) \end{array}$$

(17) $$\begin{array}{cc} \; & p\left(N^{\left(p\right)}\left(t+1\right)|N^{\left(r\right)}\left(t\right),C\left(t\right),N^{\left(p\right)}\left(t\right)\right)=\\ \; & \frac{1}{\sqrt{2\pi {\tilde{\sigma }}_{p}^{2}}}\exp\left(-\frac{{\left(N^{\left(p\right)}\left(t+1\right)-N^{\left(p\right)}\left(t\right)-\Delta_{t}\sum_{l,m,n}k_{l,m,n}^{\left(p\right)}S_{l}^{\left(p\right)}\left(N^{\left(p\right)}\left(t\right)\right){\left\{C^{m}\left(t\right)-{\left(C_{eq}^{\left(p\right)}\right)}^{m}\right\}}^{n}\right)}^{2}}{2{\tilde{\sigma }}_{p}^{2}}\right) \end{array}$$

where we put ${\tilde{\sigma }}_{r}=\sqrt{\Delta_{t}}\sigma_{r}$ and ${\tilde{\sigma }}_{p}=\sqrt{\Delta_{t}}\sigma_{p}$.

Here, we employ a sequential Monte Carlo method based on the state-space model of heterogeneous reactions. The sequential Monte Carlo method is a statistical method for estimating hidden variables of nonlinear dynamical systems by using a set of particles for posterior distributions of hidden variables [22]. We derive a method for estimating time series of hidden variables $x_{t}=[N^{\left(r\right)}\left(t\right),C\left(t\right),N^{\left(p\right)}\left(t\right)]\;\left(t=0,\cdots ,T\right)$ from the observed data $y_{0:T}=\left[y_{0},\cdots ,y_{T}\right]$ where $y_{t}=\left[C^{\prime }\left(t\right)\right]$.

The predictive distribution of a hidden variable at time step *t*, given time-series of observed data up to preceding time step t−1, $p\left(x_{t}|y_{0:t-1}\right)$, is expressed as follows [21,22,23,24]:

(18) $$\begin{array}{c} \begin{array}{c} p\left(x_{t}|y_{0:t-1}\right)=\int p\left(x_{t}|x_{t-1}\right)p\left(x_{t-1}|y_{0:t-1}\right)dx_{t-1} \end{array} \end{array}$$

where $p(x_{t}|x_{t-1})$ is the system model, and $p(x_{t-1}|y_{0:t-1})$ is the filtering distribution at time step t−1. Therefore, if we obtain the filtering distribution $p(x_{t-1}|y_{0:t-1})$ at the time step t−1, we can calculate the prediction distribution $p(x_{t}|y_{0:t-1})$ by integrating with respect to $x_{t-1}$. Moreover, the filtering distribution at time step *t*, $p\left(x_{t}|y_{0:t}\right)$, is expressed using the predictive distribution at time step t−1 as follows:

(19) $$\begin{array}{c} \begin{array}{c} p\left(x_{t}|y_{0:t}\right)=\frac{p\left(y_{t}|x_{t}\right)p\left(x_{t}|y_{0:t-1}\right)}{p\left(y_{t}|y_{0:t-1}\right)} \end{array} \end{array}$$

where $p(y_{t}|y_{0:t-1})$ is expressed as follows:

(20) $$\begin{array}{c} \begin{array}{c} p\left(y_{t}|y_{0:t-1}\right)=\int p\left(y_{t}|x_{t}\right)p\left(x_{t}|y_{0:t-1}\right)dx_{t} \end{array} \end{array}$$

Note that $p(y_{t}|x_{t})$ is the observation model. Thus, hidden variables can be estimated with forward recursion by alternatively calculating the predictive distribution and filtering distribution. Furthermore, the smoothing distribution of hidden variables at time *t*(<*T*) given the time-series of observation data up to time *T* is expressed as follows:

(21) $$\begin{array}{c} \begin{array}{c} p\left(x_{t}|y_{0:T}\right)=p\left(x_{t}|y_{0:t}\right)\int \frac{p\left(x_{t+1}|x_{t}\right)}{p\left(x_{t+1}|y_{0:t}\right)}p\left(x_{t+1}|y_{0:T}\right)dx_{t+1} \end{array} \end{array}$$

where $p(x_{t}|y_{0:t})$ indicates the filtering distribution at time step *t*. Note that the smoothing distribution at a time step *t* is obtained by that at time step t+1. Therefore, the smoothing distribution can be calculated using backward recursion.

To obtain the above conditional distributions, we express the distributions by using particle approximation. The particle approximation is employed to estimate predictive, filtering, and smoothing distributions numerically since it is difficult to conduct their updates shown in Equations (19)–(21) analytically when nonlinear dynamical systems are assumed [22]. Each conditional distribution for the hidden variables $x_{t}=[N^{\left(r\right)}\left(t\right),C\left(t\right),N^{\left(p\right)}\left(t\right)]$(t=0,⋯,T) is assumed to be expressed by many particles. In the particle approximation, the predictive distribution $p\left(x_{t}|y_{1:t-1}\right)$ and the filtering distribution $p\left(x_{t}|y_{1:t}\right)$ are expressed as follows:

(22) $$\begin{array}{c} p\left(x_{t}|y_{1:t-1}\right)≃\frac{1}{I}\sum_{i=1}^{I}\delta \left(x_{t}-x_{t|t-1}^{\left(i\right)}\right) \end{array}$$

(23) $$\begin{array}{c} p\left(x_{t}|y_{1:t}\right)≃\frac{1}{I}\sum_{i=1}^{I}\delta \left(x_{t}-x_{t|t}^{\left(i\right)}\right) \end{array}$$

where δ(t) is the Dirac’s delta function and *I* is the total number of particles. We substitute the approximated predictive and filtering distributions into Equations (18) and (19). By using the recursive relationship between the predictive and filtering distributions, we obtain a set of particles for the filtering distribution $X_{t|t}={\left[x_{t|t}^{\left(i\right)}\right]}_{i=1}^{I}$ from a set of particles for the predictive distribution $X_{t|t-1}={\left[x_{t|t-1}^{\left(i\right)}\right]}_{i=1}^{I}$, and also obtain a set of particles for the predictive distribution $X_{t+1|t}$ from that for the filtering distribution $X_{t|t}$. Based on the sequential Monte Carlo method, we estimate hidden variables by using particles ${\left[x_{t|t}^{\left(i\right)}\right]}_{i=1}^{I}=[N_{i}^{\left(r\right)}\left(t\right),C_{i}\left(t\right),N_{i}^{\left(p\right)}{\left(t\right)]}_{i=1}^{I}$ from the partially observed data $y_{t}=\left[C^{\prime }\left(t\right)\right]$.

### 2.3. Sparse Modeling Algorithm for Estimating Rate Constants

Rate constants $k=\left\{k_{l,m,n}^{\left(r\right)},k_{l,m,n}^{\left(p\right)}\right\}$ play a important role as determinants for reaction rates and surface-area reactions. Moreover, rate constants determine linearity or nonlinearity of reaction term ${\left\{C^{m}-{\left(C_{eq}\right)}^{m}\right\}}^{n}$ and intermediate product *C* in Equations (1) and (2). According to Equations (1) and (2), there are $\tilde{l}\times \tilde{m}\times \tilde{n}$ types of reactant and product reactions. Therefore, there are $2^{2\times \tilde{l}\times \tilde{m}\times \tilde{n}}$ reaction candidates of heterogeneous reactions.

We employ a sparse modeling approach in order to estimate rate constants $k=\left\{k_{l,m,n}^{\left(r\right)},k_{l,m,n}^{\left(p\right)}\right\}$ from many candidates. The sparse modeling is a framework for extracting only essential elements from candidates by assuming that the essential elements are *sparse* compared with a number of candidates [25,26]. Based on Equations (1) and (2), there are $2^{2\times \tilde{l}\times \tilde{m}\times \tilde{n}}$ reaction candidates. By using the sparse modeling approach, we estimate each value of rate constants $k=\left\{k_{l,m,n}^{\left(r\right)},k_{l,m,n}^{\left(p\right)}\right\}$ and determine as zero unnecessary terms or non-zero for necessary terms. Then, we extract only the necessary heterogeneous reactions.

**Figure 5:**
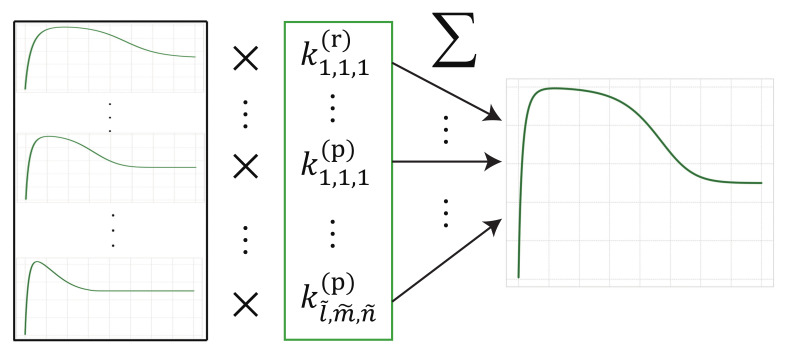
Conceptual diagram of sparse modeling approach for nonlinear dynamics in heterogeneous reactions. The nonlinear differential equation for heterogeneous reactions (Equations (10)–(12)) can be expressed by as a linear sum of the candidate reaction terms. The proposed method, using the sparse modeling approach, extracts essential reaction terms from candidates by estimating the rate constants $k_{l,m,n}^{\left(r\right)}$ and $k_{l,m,n}^{\left(p\right)}$: zero and non-zero values for unnecessary and necessary terms, respectively.

Figure 5 shows the schematic diagram of the estimation method for surface-area reaction in heterogeneous reactions by sparse modeling. The difference equations for heterogeneous reactions can be regarded as the following linear equation with respect to the rate constants $k_{l,m,n}^{\left(r\right)},k_{l,m,n}^{\left(p\right)}$; each heterogeneous reaction is multiplied by the reaction rate coefficient $k_{l,m,n}^{\left(r\right)},k_{l,m,n}^{\left(p\right)}$ as follows:

(24) $$\begin{array}{cc} -\left(C(t+1)-C(t)\right)=\Delta_{t} & \sum_{l=1}^{\tilde{l}}\sum_{m=1}^{\tilde{m}}\sum_{n=1}^{\tilde{n}}k_{l,m,n}^{\left(r\right)}S_{l}^{\left(r\right)}\left(N^{\left(r\right)}\left(t\right)\right){\left\{C^{m}\left(t\right)-{\left(C_{eq}^{\left(r\right)}\right)}^{m}\right\}}^{n}\\ +\Delta_{t} & \sum_{l=1}^{\tilde{l}}\sum_{m=1}^{\tilde{m}}\sum_{n=1}^{\tilde{n}}k_{l,m,n}^{\left(p\right)}S_{l}^{\left(p\right)}\left(N^{\left(p\right)}\left(t\right)\right){\left\{C^{m}\left(t\right)-{\left(C_{eq}^{\left(p\right)}\right)}^{m}\right\}}^{n} \end{array}$$

Notably, the right-hand side of Equation (24) is expressed as a linear sum of the reaction term $\left\{S{}_{l}\left(N_{i}\left(t\right)\right)\right\}{\left\{C_{i}^{m}\left(t\right)-{\left(C_{eq}\right)}^{m}\right\}}^{n}$ with coefficients $k_{l,m,n}$ corresponding to rate constants. To extract rate constant terms from a number of candidates, we use the Lasso framework (least absolute shrinkage and selection operator), which employs a sparse modeling approach [6,7,27,28]. By considering the $L_{1}$ regularization term for rate constants, sparse modeling of heterogeneous reactions is formulated based on the Lasso framework as follows:

(25) $$\begin{array}{c} \underset{k}{\mathrm{argmin}}\left|\right|z_{1:I,0:T}-A_{1:I,0:T}{k||}_{2}^{2}+{\lambda ||k||}_{1} \end{array}$$

(26) $$\begin{array}{cc} {\left||k|\right|}_{1} & =|k_{1,1,1}^{\left(r\right)}\left|+\cdots +\right|k_{1,1,1}^{\left(p\right)}\left|+\cdots +\right|k_{\tilde{l},\tilde{m},\tilde{n}}^{\left(p\right)}| \end{array}$$

where $z_{1:I,0:T}$, $A_{1:I,0:T}$ and k are represented by the following matrix and vectors:

(27) $$\begin{array}{ccc} z_{1:I,0:T} & = & \frac{1}{I}\sum_{i=1}^{I}\left[\begin{array}{c} -C_{i}\left(1\right)+C_{i}\left(0\right)\\ -C_{i}\left(2\right)+C_{i}\left(1\right)\\ \vdots \\ -C_{i}\left(T\right)+C_{i}\left(T-1\right) \end{array}\right]=\left[\begin{array}{c} \bar{-C(1)+C(0)}\\ \bar{-C(2)+C(1)}\\ \vdots \\ \bar{-C(T)+C(T-1)} \end{array}\right] \end{array}$$

(28) $$\begin{array}{c} A_{1:I,0:T}=\Delta_{t}\left[\begin{array}{ccc} \bar{S_{1}^{\left(r\right)}\left(N^{\left(r\right)}\left(0\right)\right)\left\{C\left(0\right)-\left(C_{eq}^{\left(r\right)}\right)\right\}} & \cdots & \bar{S_{\tilde{l}}^{\left(p\right)}\left(N^{\left(p\right)}\left(0\right)\right){\left\{C^{\tilde{m}}\left(0\right)-{\left(C_{eq}^{\left(p\right)}\right)}^{\tilde{m}}\right\}}^{\tilde{n}}}\\ \;\bar{S_{1}^{\left(r\right)}\left(N^{\left(r\right)}\left(1\right)\right)\left\{C\left(1\right)-\left(C_{eq}^{\left(r\right)}\right)\right\}} & \cdots & \bar{S_{\tilde{l}}^{\left(p\right)}\left(N^{\left(p\right)}\left(1\right)\right){\left\{C^{\tilde{m}}\left(1\right)-{\left(C_{eq}^{\left(p\right)}\right)}^{\tilde{m}}\right\}}^{\tilde{n}}}\\ \;\vdots & \ddots & \vdots \\ \bar{S_{1}^{\left(r\right)}\left(N^{\left(r\right)}\left(T-1\right)\right)\left\{C\left(T-1\right)-\left(C_{eq}^{\left(r\right)}\right)\right\}} & \cdots & \bar{S_{\tilde{l}}^{\left(p\right)}\left(N^{\left(p\right)}\left(T-1\right)\right){\left\{C^{\tilde{m}}\left(T-1\right)-{\left(C_{eq}^{\left(p\right)}\right)}^{\tilde{m}}\right\}}^{\tilde{n}}}\; \end{array}\right] \end{array}$$

(29) $$\begin{array}{ccc} k & = & \left[\begin{array}{c} k_{1,1,1}^{\left(r\right)}\\ \vdots \\ k_{1,1,1}^{\left(p\right)}\\ \vdots \\ k_{\tilde{l},\tilde{m},\tilde{n}}^{\left(p\right)} \end{array}\right] \end{array}$$

In Equation (25), λ is the regularization coefficient that controls the relative importance of a data-dependent error $\left|\right|z_{1:I,0:T}-A_{1:I,0:T}k{\left|\right|}_{2}^{2}$ and the regularization term ${\left||k|\right|}_{1}$. The regularization term ${\left||k|\right|}_{1}$ is the sum of the absolute values of each rate constants $|k_{l,m,n}|$. When the regularization term ${\left||k|\right|}_{1}$ becomes smaller, the rate constants approach zero, and we can obtain a sparse solution. Here, $z_{1:I,0:T}$ is a vector consisting of the averages of the differences $\bar{-C(t+1)+C(t)}$ between intermediate products’ adjacent time steps, which are obtained by particles as the averages of the difference $-C_{i}\left(t+1\right)+C_{i}\left(t\right)$(i=1,⋯,I). $A_{1:I,0:T}$ denotes a matrix consisting of the average of reaction terms, which are obtained by particles (i=1,⋯,I). k indicates a vector consisting of rate constants. We calculate the matrix $A_{1:I,0:T}$ and vector $z_{1:I,0:T}$ by using hidden variables $[N_{i}^{\left(r\right)}{]}_{i=1}^{I}$, ${\left[C_{i}\right]}_{i=1}^{I}$ and $[N_{i}^{\left(p\right)}{]}_{i=1}^{I}$. To obtain an appropriate value of λ, we employ nested cross-validation error since time-series data are used [29].

To compare with the Lasso framework, we consider the Ridge framework, expressed by the following equation [30]:

(30) $$\underset{k}{\mathrm{argmin}}\left|\right|z_{1:I,0:T}-A_{1:I,0:T}{k||}_{2}^{2}+{\lambda ||k||}_{2}^{2}$$

(31) $${\left||k|\right|}_{2}={\left(|k_{1,1,1}^{\left(r\right)}{|}^{2}+\cdots +|k_{1,1,1}^{\left(p\right)}{|}^{2}+\cdots +|k_{\tilde{l},\tilde{m},\tilde{n}}^{\left(p\right)}{|}^{2}\right)}^{1/2}$$

**Figure 6:**
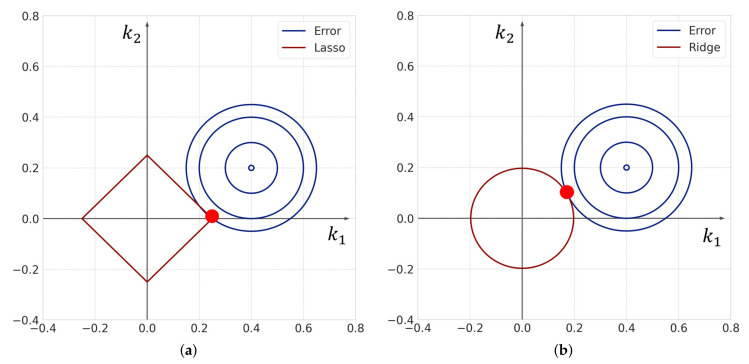
Conceptual diagram of (**a**) Lasso and (**b**) Ridge frameworks in two dimensions. The red dot in each figure indicates the estimated values in the corresponding algorithm. A sparse solution is obtained in the case of Lasso, whereas a non-sparse solution is obtained in the case of Ridge.

Namely, the Ridge framework introduces the $L_{2}$ regularization term. Ridge and Lasso frameworks are different in terms of regularization terms. Figure 6 shows a conceptual diagram of the Lasso and Ridge frameworks in two dimensions. Lasso yields a sparse solution because the part where the contour line of the error term and the boundary of the constraint condition meet is likely to be a corner since the constraint condition of Lasso is given by ${\left||k|\right|}_{1}$. In Figure 6, $k_{2}$ is estimated to be a zero value by using Lasso. However, when Ridge is used, both $k_{1}$ and $k_{2}$ are estimated to be non-zero values. Therefore, the Lasso framework can obtain a sparse solution more effectively than the Ridge framework. Thus, we assume that the sparse modeling approach extracts only essential reactions from candidates in heterogeneous reactions by estimating each rate constant as either zero or non-zero.

However, in the case where we can observe few data or many candidates can be assumed, it may not be possible to extract only important reactions. Therefore, instead of uniform sparsity levels, we introduce non-uniform ones in order to extract only the important reactions more accurately. Here, a sparse modeling approach based on adaptive Lasso is introduced [31]. The sparse modeling approach with non-uniform sparsity levels is expressed by the following equation:

(32) $$\underset{k}{\mathrm{argmin}}\left|\right|z_{1:I,0:T}-A_{1:I,0:T}{k||}_{2}^{2}+\lambda \sum_{j=1}^{2\tilde{l}\tilde{m}\tilde{n}}\omega_{j}\left|k_{j}\right|$$

(33) $$\omega_{j}=\frac{1}{|\beta_{j}{|}^{\gamma }}$$

where $\beta =\{\beta_{j}\}$ is a consistent estimator, and it is determined by the least-squares method and the Ridge framework. Here, $k_{j}$ is *j*-th element of vector k shown in Equation (29). According to Equation (33), $\omega_{j}$ is the reciprocal of the absolute value of $\beta_{j}$ to the γ(γ>0) power. Here, we employ the Ridge framework represented by Equation (30) to obtain β and put γ=1. If the value of $|\beta_{j}|$ is small, the values of $|\omega_{j}|$ become large. Therefore, the sparsity tends to increase to realize $|k_{j}|=0$. In contrast, if the value of $|\beta_{j}|$ is large, the value of $|\omega_{j}|$ becomes small, and the sparsity tends to decrease to realize $|k_{j}|\neq 0$.

**Figure 7:**
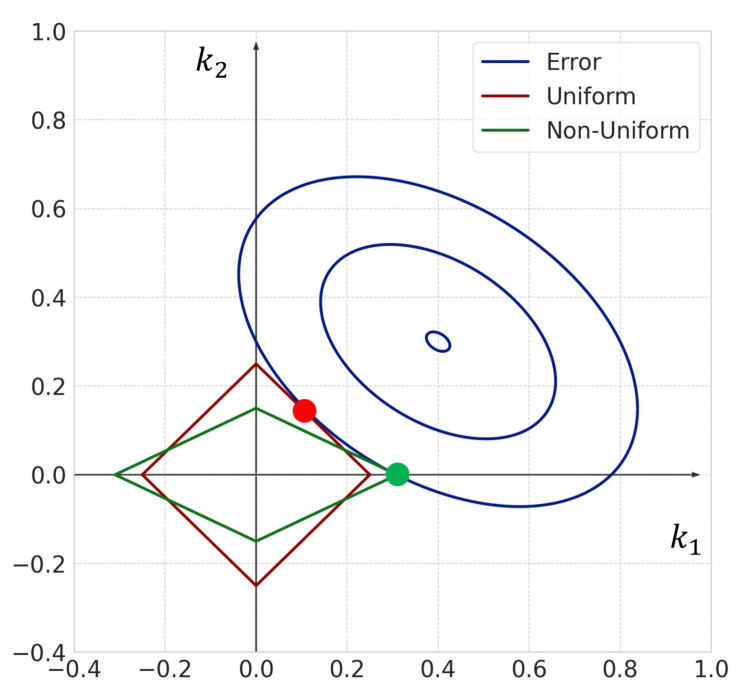
Conceptual diagram of sparse modeling with uniform and non-uniform sparsity levels. A sparse solution is obtained using sparse modeling with non-uniform sparsity levels.

Figure 7 shows a conceptual diagram of the sparse modeling approach with uniform and non-uniform sparsity levels for a two-dimensional vector. For the sparse modeling approach with non-uniform sparsity levels, the regularization term has non-uniformity, due to the weight vector obtained by the consistent estimator. The intersection of the contour line of the error term and regularization term of non-uniform sparsity is $\left(k_{1},k_{2}\right)=\left({k_{1}}^{\prime },0\right)$. Therefore, non-uniform sparsity yields a sparse solution. However, for the sparse modeling approach with uniform sparsity levels, the intersection of the contour line of the error term and regularization term of uniform sparsity is $\left(k_{1},k_{2}\right)=\left({k_{1}}^{\prime },{k_{2}}^{\prime }\right)$ where both elements of the weight vector become non-zero (${k_{1}}^{\prime }\neq 0$ and ${k_{2}}^{\prime }\neq 0$). Therefore, sparse modeling with uniform sparsity may not yield a sparse solution. Thus, we employ the sparse modeling approach with non-uniform sparsity levels in order to extract only the important reactions more accurately.

## 3. Results

In this section, we evaluate the effectiveness of our proposed method using simulation data. The proposed method is a combined method based on sparse modeling and sequential Monte Carlo approaches. Multi-dimensional hidden variables consisting of solid reactant $N^{\left(r\right)}$, liquid intermediate product *C*, and solid product $N^{\left(p\right)}$ are estimated from a partially observed liquid intermediate product $C^{\prime }$ by using the sequential Monte Carlo method. We employ the sparse modeling approach to estimate the rate constants $k=\left\{k_{l,m,n}^{\left(r\right)},k_{l,m,n}^{\left(p\right)}\right\}$. By conducting these two calculations alternately, we simultaneously estimate $N^{\left(r\right)},C,N^{\left(p\right)}$ and $k=\left\{k_{l,m,n}^{\left(r\right)},k_{l,m,n}^{\left(p\right)}\right\}$.

We assume that the data $C^{\prime }\left(t\right)$ are available at constant time step intervals within total time steps *T*. In Equation (8), the observation noise $\xi^{C}\left(t\right)$ is assumed to be a Gaussian noise with zero means and standard deviation $1\times 10^{-3}$. Further, in Equations (13>) and (14), the system noise $\xi^{(}r)\left(t\right)$ and $\xi^{(}p)\left(t\right)$ are also assumed to be Gaussian noise with zero mean and standard deviation $1\times 10^{-4}$. Since the reaction time is 0≤t≤4000 and the time interval is $\Delta_{t}=1$, the maximum number of observed data is 4001 points. We set $\tilde{l}=3,\tilde{m}=2,\tilde{n}=2$ in Equations (13) and (14). Note that $\tilde{l}$ denotes the number of candidates for the type of surface-area model, $\tilde{m}$ is the number of candidates for a multiplier of the intermediate product *C*, and $\tilde{n}$ is the number of candidates for a multiplier of the reaction term $\left\{C^{m}-{\left(C_{eq}\right)}^{m}\right\}$. Therefore, the number of rate constants k is $2\times \tilde{l}\times \tilde{m}\times \tilde{n}=2\times 3\times 2\times 2=24$ and we assume $2^{24}$ nonlinear or linear candidates. The value of each parameter used to obtain the simulation data is shown in Table 1.

We set the initial values of the hidden variables $N^{\left(r\right)}\left(0\right)=0.99$, C(0)=0.005, $N^{\left(p\right)}\left(0\right)=0.005$ and the initial values of rate constants k random values near zero. From Equations (13)–(15) and Table 1, we generate the simulation data, and the reaction is consistent with the typical heterogeneous reaction dynamics shown in Figure 3. We employ the sparse modeling and sequential Monte Carlo method approaches alternately for a sufficient number of times, and use values with which the generalization error, using cross-validation, is the smallest as the estimated results.

### 3.1. Simultaneous Estimation of Hidden Variables and Rate Constant

**Figure 8:**
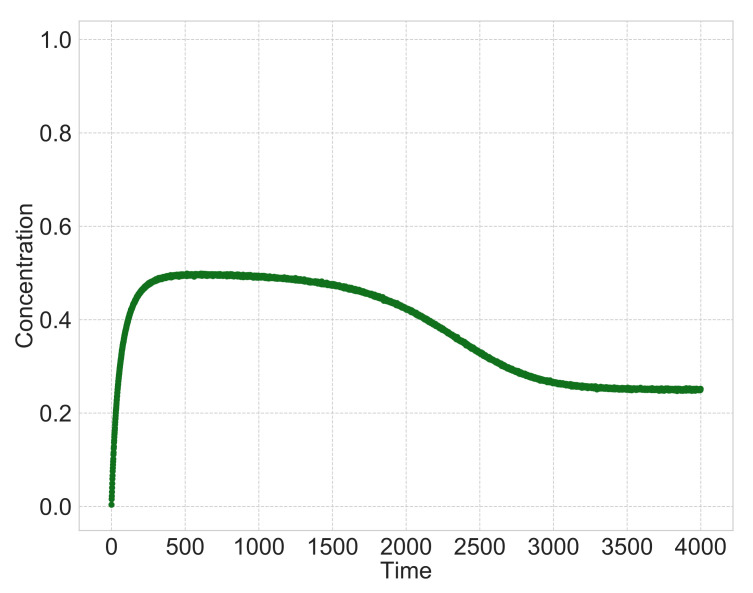
Observation variable $C^{\prime }\left(t\right)$ for observation points T=4000.

Here, we employ the proposed method to estimate the hidden variables and the rate constants by using 4000 observation points. The observation values under the conditions are shown in Figure 8. We employ the sparse modeling with uniform sparsity levels and that with non-uniform sparsity levels, which are our proposed methods, in order to estimate rate constants k. We compare results with those of two other methods: Ridge in Equation (30) and the least-squares method.

**Figure 9:**
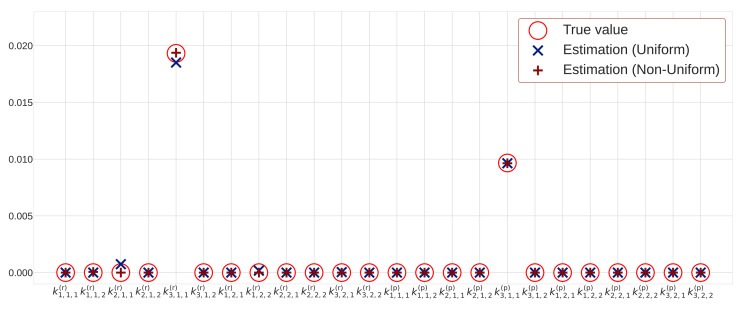
Estimated rate constants for observation points T=4000.

**Figure 10:**
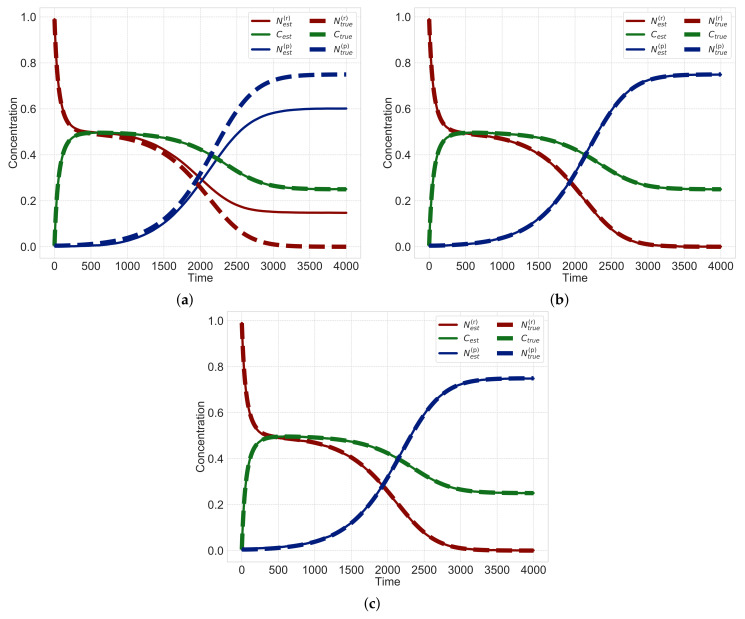
Estimated hidden variables $N^{\left(r\right)}$, *C* and $N^{\left(p\right)}$ for data points T=4000. The dotted and solid lines represent the true and estimated values, respectively. (**a**) Ridge; (**b**) Uniform sparsity; (**c**) Non-uniform sparsity.

Table 2 lists the estimation results of each rate constants obtained using different methods. We find that by using the least-squares method, the estimated values based on the sequential Monte Carlo method diverge. Note that the values for the least-squares method listed in Table 2 are estimated rate constants just before the values diverge. The reaction rate constants estimated by sparse modeling with uniform sparsity levels and that with non-uniform sparsity levels are also shown in Figure 9. Figure 10 shows the estimated hidden variables based on the sequential Monte Carlo method obtained by using the estimated rate constants in Table 2. Table 2 also shows the mean squared error (MSE) between the estimated hidden variables and simulation data.

First, we evaluate the estimation result of the rate constants. Table 2 shows that the rate constants estimated by the least squares method are quite different from true values. Although the rate constants estimated with Ridge does not deviate as much as those with the least-squares method, they have many non-zero rate constants. Therefore, it is difficult to determine which reaction is the most important. On the other hand, the proposed methods based on uniform sparsity levels and non-uniform sparsity levels can obtain many zero values for rate constants, and it is possible to extract important reactions. Here, we compare the two sparse modeling methods regarding the number of non-zero rate constants. Table 2 reveals that the method with non-uniform sparsity levels provides three non-zero elements, whereas the method with uniform sparsity levels provides five non-zero elements. Therefore, we find that the proposed method with non-uniform sparsity levels can extract important reactions better than that with uniform sparsity levels. Notably, the proposed method can be applied to other cases with different true reaction terms in order to extract only essential reaction terms from the candidate reaction terms.

Next, we evaluate hidden variables estimated by using the sequential Monte Carlo method based on the obtained rate constants. In Figure 10, the dotted and solid lines represent the true and estimated values, respectively. Since the dotted and solid lines in Figure 10a do not align, hidden variables cannot be well-estimated using Ridge. In contrast, the dotted and solid lines in the results obtained from sparsity modeling with uniform sparsity levels and non-uniform sparsity levels almost overlap (Figure 10b,c). These results imply that the proposed method can estimate three hidden variables $N^{\left(r\right)}$, *C* and $N^{\left(p\right)}$ from observed data $C^{\prime }$. We also evaluate the mean squared error (MSE) in Table 2. Since the MSE obtained from the method with uniform sparsity levels is larger than that with non-uniform sparsity levels, the estimated results obtained from the method with non-uniform sparsity levels are found to be better than those with uniform sparsity levels.

Therefore, the proposed method can simultaneously estimate three hidden variables $N^{\left(r\right)}$, *C* and $N^{\left(p\right)}$ as well as rate constants $k=\left\{k_{l,m,n}^{\left(r\right)}k_{l,m,n}^{\left(p\right)}\right\}$. Moreover, the sparse modeling method with non-uniform sparsity levels is better than that with uniform sparsity levels in estimating hidden variables and rate constants.

### 3.2. Simultaneous Estimation of Hidden Variables and Rate Constant for a Small Number of Observation Points

**Figure 11:**
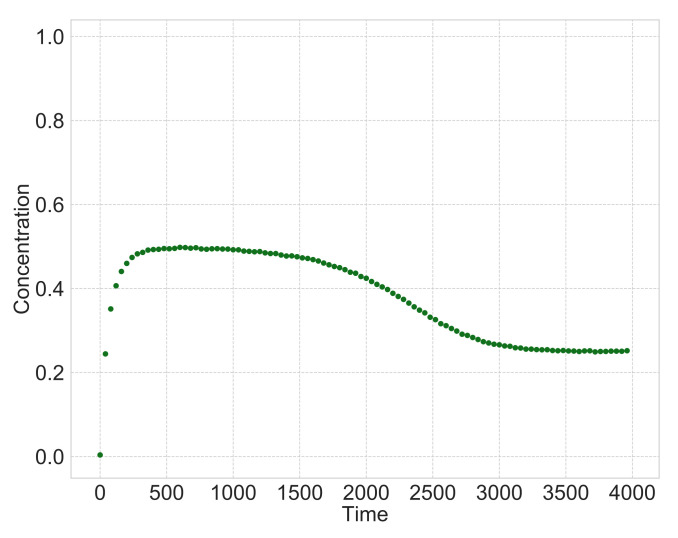
Observation variable $C^{\prime }\left(t\right)$ for observation points T=100.

**Figure 12:**
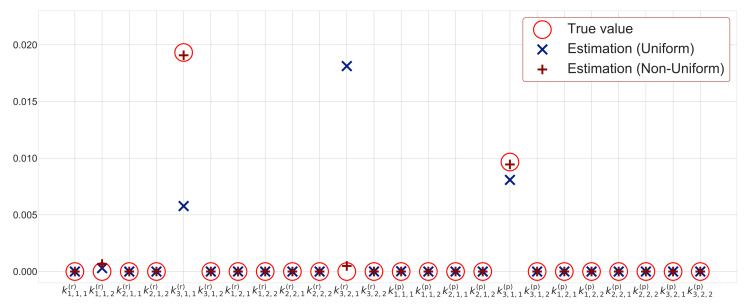
Estimated rate constants for observation points T=100.

**Figure 13:**
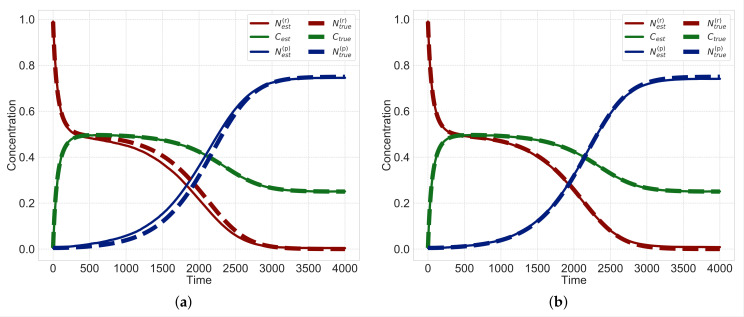
Estimated hidden variables for the number of observation points T=100. The dotted lines represent true values and solid lines represent estimated values. (**a**) Uniform sparsity; (**b**) Non-uniform sparsity.

Here, we consider a situation where the number of observation points is limited. We validate the proposed method under the condition that the number of observation points is less than that in Section 3.1. We set the number of observation points to 100. The observation values under the conditions are shown in Figure 11. The number of observation points is small and sparse compared to that in Figure 8, which has 4000 observation points. We estimate heterogeneous reactions using the proposed method with uniform and non-uniform sparsity levels in order to estimate the rate constants k. The estimated rate constants are shown in Table 3. The reaction rate constants estimated using the method with uniform sparsity levels and that with non-uniform sparsity levels are also shown in Figure 12. We further use estimated rate constants in Table 3 to calculate the hidden variables based on the sequential Monte Carlo method, and the results are shown in Figure 13. Additionally, Table 3 shows the MSE between the estimated hidden variables and simulation data.

First, we evaluate the estimation result of the rate constants. From Figure 12, non-zero values of rate constants estimated by uniform sparsity show $k_{3,2,1}^{\left(r\right)}$, $k_{3,2,1}^{\left(r\right)}$ and $k_{3,1,1}^{\left(p\right)}$ in descending order. However, the rate constant $k_{3,2,1}^{\left(r\right)}$ must be zero. Therefore, the method with uniform sparsity levels cannot estimate rate constants accurately when the number of observation points is 100. On the other hand, the proposed methods based on non-uniform sparsity levels can well estimate the rate constants at such a condition since the true and estimated values almost overlap, as shown in Figure 12. Additionally, we compare the proposed two sparse modeling methods in terms of the number of non-zero values of the rate constants. Table 3 shows that the method with non-uniform sparsity levels provides four non-zero elements, and that with uniform sparsity levels provides five non-zero elements. Therefore, even when the number of observation points is small, the method with non-uniform sparsity levels can extract important reactions better than that with uniform sparsity levels.

Next, we evaluate the hidden variables estimated by using the sequential Monte Carlo method, based on the estimated rate constants. Since time courses of the estimated variables (the dotted line) and that of true variables (the solid line) are slightly different in Figure 13a, we find that the proposed method with uniform sparsity levels cannot well estimate hidden variables. In contrast, as shown in Figure 13b, the estimation results of the proposed method with non-uniform sparsity levels show that the time courses of the estimated variables (dotted line) and true variables (solid line) almost overlap. These results show that the proposed method with non-uniform sparsity levels can estimate three hidden variables $N^{\left(r\right)}$, *C* and $N^{\left(p\right)}$ from observed data $C^{\prime }$. We then evaluate the MSE in Table 3. Since the MSE of the method with uniform sparsity levels is higher than that with non-uniform sparsity levels, we see that the method with non-uniform sparsity levels is better than that with uniform sparsity levels when the number of observation points is small.

These results show that even when the number of observation points is small, the proposed method with non-uniform sparsity levels can estimate simultaneously three hidden variables $N^{\left(r\right)}$, *C* and $N^{\left(p\right)}$ and rate constants $k=\left\{k_{l,m,n}^{\left(r\right)}k_{l,m,n}^{\left(p\right)}\right\}$.

### 3.3. Dependence of the Estimation Accuracy on the Number of Observation Points

**Figure 14:**
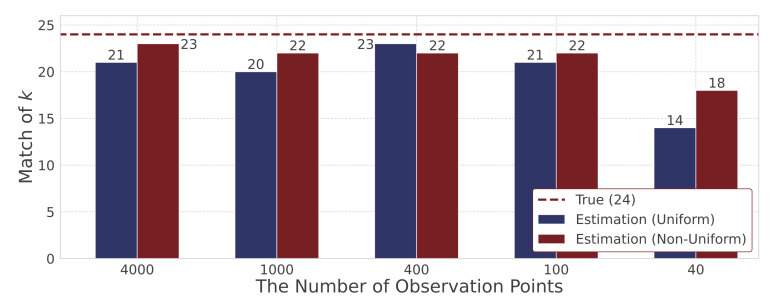
The degree of non-zero and zero agreement for different numbers of observation points.

**Figure 15:**
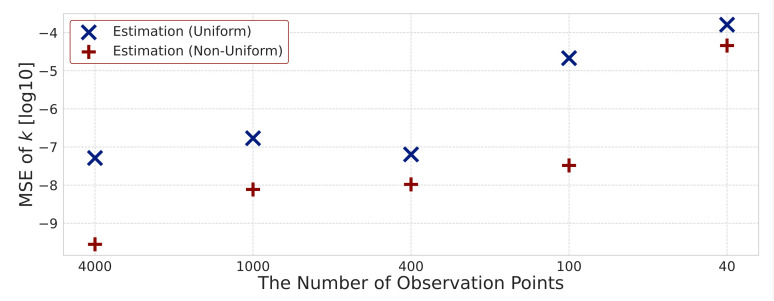
Mean squared error between true and estimated rate constants for different numbers of observation points.

**Figure 16:**
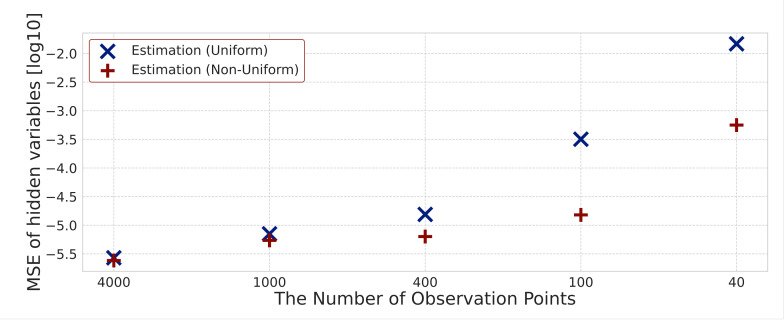
Mean squared error between true and estimated hidden variables for different numbers of observation points.

Here, we investigate how accurately the rate constants $k=\left\{k_{l,m,n}^{\left(r\right)},k_{l,m,n}^{\left(p\right)}\right\}$ can be estimated for different numbers of observations. We estimate the rate constants for different numbers of observation points, including T=4000, 1000, 400, 100 and 40, to consider the effectiveness of the proposed method with uniform sparsity levels and that with non-uniform sparsity levels. We focus on the difference between the true and estimated rate constants. Figure 14 shows the degree of non-zero and zero agreement, and Figure 15 shows the MSE. Since we assume 24 kinds of rate constants, the maximum degree of agreement is 24. Figure 16 shows the MSE between the estimated hidden variables and simulation data for different numbers of observation points. Both MSE in Figure 15 and Figure 16 are expressed using the logarithms to base 10.

First, we evaluate the estimated rate constants. Figure 14 shows that when the number of observation points is varied from 4000 to 100, there is not much difference in the degree of agreement of rate constants between the proposed method with non-uniform sparsity levels and that with uniform sparsity levels. This result indicates that we can select non-zero and zero rate constants. Therefore, we can extract important reactions from many candidates when the number of observable points is sufficiently large by means of both the proposed methods. On the other hand, Figure 15 shows that the proposed method with non-uniform sparsity levels has a smaller MSE of rate constants than that with uniform sparsity levels for all observation points. Figure 14 and Figure 15 reveal that the proposed method with non-uniform sparsity levels shows small discrepancy between the estimated and true rate constants $k=\left\{k_{l,m,n}^{\left(r\right)},k_{l,m,n}^{\left(p\right)}\right\}$ compared with that with uniform sparsity levels. When the number of observation points is 40, both methods have low accuracy in the degree of coincidence and the MSE. Therefore, it is difficult to estimate rate constants when the number of observation points is 40, and we consider around 100 points as the limit to estimate rate constants when candidates of heterogeneous reaction terms assumed in our study are given.

Next, we consider the MSE between the simulation data and estimated hidden variables. Figure 16 shows that the MSE increases as the number of observation points decreases with both non-uniform and uniform sparsity levels. This result indicates that the proposed method can estimate hidden variables more accurately as the number of observation points increases. In addition, as the number of observation points decreases, the difference in the MSE between the two methods increases. Therefore, we find that when the number of observation points decreases, the proposed method using non-uniform sparsity levels is more effective.

To summarize, the proposed method with non-uniform sparsity levels can estimate rate constants accurately from partially observable data; Using time series of observed data points of an intermediate product, we have shown that the proposed method can extract essential reaction terms from candidates; the rate constants of necessary terms are estimated to be non-zeros, whereas those of unnecessary terms are estimated to be zeros.

## 4. Concluding Remarks

We have proposed an algorithm based on the sparse modeling and sequential Monte Carlo approaches for estimating heterogeneous reaction dynamics. We focused on heterogeneous reactions, which depend on the surface-area reactions between solid and liquid phases, and we developed a nonlinear state-space model for the heterogeneous reactions. We considered three aspects: *l*$\left(l=1,2,\cdots ,\tilde{l}\right)$ types of surface-area reactions, the multiplier of the intermediate product $C^{m}$$\left(m=1,2,\cdots ,\tilde{m}\right)$ and the multiplier of the reaction term ${\left\{C^{m}-{\left(C_{eq}\right)}^{m}\right\}}^{n}$$\left(n=1,2,\cdots \tilde{n}\right)$. The results in this study showed that the proposed sparse modeling method can extract essential reaction terms from candidates; the rate constants of necessary terms are estimated to be non-zeros and those of unnecessary terms are estimated to be zeros. By introducing the sequential Monte Carlo algorithm, we estimated the multi-dimensional hidden variables consisting of the solid reactant, liquid intermediate product, and solid product from the partially observed data of the liquid intermediate product. The results have shown that the proposed method with non-sparsity levels simultaneously estimates the time course of hidden variables and rate constants accurately by extracting sufficient reaction terms from the candidates.

The following abbreviations are used in this manuscript:

## Figures and Tables

**Table 1:** Parameter values for simulations.

Parameter	Value	Parameter	Value
$C_{eq}^{\left(r\right)}$	0.5	$C_{eq}^{\left(p\right)}$	0.25
$k_{1,1,1}^{\left(r\right)}$	0	$k_{1,1,1}^{\left(p\right)}$	0
$k_{1,1,2}^{\left(9\right)}$	0	$k_{1,1,2}^{\left(p\right)}$	0
$k_{2,1,1}^{\left(r\right)}$	0	$k_{2,1,1}^{\left(p\right)}$	0
$k_{2,1,2}^{\left(r\right)}$	0	$k_{2,1,2}^{\left(p\right)}$	0
$k_{3,1,1}^{\left(p\right)}$	1.93 × 10${}^{-2}$	$k_{3,1,1}^{\left(p\right)}$	9.66 × 10${}^{-3}$
$k_{3,1,2}^{\left(r\right)}$	0	$k_{3,1,2}^{\left(p\right)}$	0
$k_{1,2,1}^{\left(r\right)}$	0	$k_{1,2,1}^{\left(p\right)}$	0
$k_{1,2,2}^{\left(r\right)}$	0	$k_{1,2,2}^{\left(p\right)}$	0
$k_{2,2,1}^{\left(r\right)}$	0	$k_{2,2,1}^{\left(p\right)}$	0
$k_{2,2,2}^{\left(r\right)}$	0	$k_{2,2,2}^{\left(p\right)}$	0
$k_{3,2,1}^{\left(r\right)}$	0	$k_{3,2,1}^{\left(p\right)}$	0
$k_{3,2,2}^{\left(r\right)}$	0	$k_{3,2,2}^{\left(p\right)}$	0

**Table 2:** Estimated rate constants for the number of data points T=4000.

	True Value	Least Squares	Ridge	Uniform	Non-Uniform
MSE		—	$5.55\times 10^{-3}$	$2.70\times 10^{-6}$	$2.42\times 10^{-6}$
$k_{1,1,1}^{\left(r\right)}$	0	$-5.39\times 10^{8}$	$-2.85\times 10^{-3}$	0	0
$k_{1,1,2}^{\left(r\right)}$	0	$1.72\times 10^{9}$	$-1.86\times 10^{-4}$	0	$5.82\times 10^{-5}$
$k_{2,1,1}^{\left(r\right)}$	0	$4.91\times 10^{8}$	$6.95\times 10^{-3}$	$7.40\times 10^{-4}$	0
$k_{2,1,2}^{\left(r\right)}$	0	$4.91\times 10^{8}$	$-2.02\times 10^{-3}$	0	0
$k_{3,1,1}^{\left(r\right)}$	1.93 × 10${}^{-2}$	$-8.04\times 10^{8}$	$8.28\times 10^{-3}$	1.85 × 10${}^{-2}$	1.94 × 10${}^{-2}$
$k_{3,1,2}^{\left(r\right)}$	0	$-8.04\times 10^{8}$	$-2.28\times 10^{-3}$	0	0
$k_{1,2,1}^{\left(r\right)}$	0	$1.29\times 10^{9}$	$-3.03\times 10^{-3}$	0	0
$k_{1,2,2}^{\left(r\right)}$	0	$-2.53\times 10^{7}$	$4.94\times 10^{-4}$	$1.70\times 10^{-4}$	0
$k_{2,2,1}^{\left(r\right)}$	0	$-4.91\times 10^{8}$	$4.93\times 10^{-3}$	0	0
$k_{2,2,2}^{\left(r\right)}$	0	5.74	$-7.74\times 10^{-4}$	0	0
$k_{3,2,1}^{\left(r\right)}$	0	$8.04\times 10^{8}$	$6.00\times 10^{-3}$	$4.58\times 10^{-5}$	0
$k_{3,2,2}^{\left(r\right)}$	0	−6.28	$-9.66\times 10^{-4}$	0	0
$k_{1,1,1}^{\left(p\right)}$	0	$4.12\times 10^{9}$	$5.21\times 10^{-4}$	0	0
$k_{1,1,2}^{\left(p\right)}$	0	$3.73\times 10^{9}$	$-7.67\times 10^{-4}$	0	0
$k_{2,1,1}^{\left(p\right)}$	0	$3.59\times 10^{8}$	$2.63\times 10^{-3}$	0	0
$k_{2,1,2}^{\left(p\right)}$	0	$7.18\times 10^{8}$	$-1.60\times 10^{-4}$	0	0
$k_{3,1,1}^{\left(p\right)}$	9.66 × 10${}^{-3}$	$3.51\times 10^{7}$	$2.84\times 10^{-3}$	9.64 × 10${}^{-3}$	9.64 × 10${}^{-3}$
$k_{3,1,2}^{\left(p\right)}$	0	7.01 × 10${}^{7}$	$4.75\times 10^{-5}$	0	0
$k_{1,2,1}^{\left(p\right)}$	0	$-6.74\times 10^{9}$	$-5.06\times 10^{-4}$	0	0
$k_{1,2,2}^{\left(p\right)}$	0	$2.53\times 10^{7}$	$-1.69\times 10^{-4}$	0	0
$k_{2,2,1}^{\left(p\right)}$	0	$-7.18\times 10^{8}$	$1.15\times 10^{-3}$	0	0
$k_{2,2,2}^{\left(p\right)}$	0	$6.67\times 10^{-1}$	$-1.58\times 10^{-4}$	0	0
$k_{3,2,1}^{\left(p\right)}$	0	$-7.01\times 10^{7}$	$1.47\times 10^{-3}$	0	0
$k_{3,2,2}^{\left(p\right)}$	0	−1.94	$-3.80\times 10^{-5}$	0	0

**Table 3:** Estimated rate constants for observation points T=100.

	True Value	Uniform	Non-Uniform
MSE		$3.18\times 10^{-4}$	$1.52\times 10^{-5}$
$k_{1,1,1}^{\left(r\right)}$	0	0	0
$k_{1,1,2}^{\left(r\right)}$	0	$3.02\times 10^{-4}$	$6.63\times 10^{-4}$
$k_{2,1,1}^{\left(r\right)}$	0	0	0
$k_{2,1,2}^{\left(r\right)}$	0	0	0
$k_{3,1,1}^{\left(r\right)}$	1.93 × 10${}^{-2}$	5.77 × 10${}^{-3}$	1.91 × 10${}^{-2}$
$k_{3,1,2}^{\left(r\right)}$	0	0	0
$k_{1,2,1}^{\left(r\right)}$	0	0	0
$k_{1,2,2}^{\left(r\right)}$	0	0	0
$k_{2,2,1}^{\left(r\right)}$	0	0	0
$k_{2,2,2}^{\left(r\right)}$	0	0	0
$k_{3,2,1}^{\left(r\right)}$	0	$1.81\times 10^{-2}$	$4.90\times 10^{-4}$
$k_{3,2,2}^{\left(r\right)}$	0	0	0
$k_{1,1,1}^{\left(p\right)}$	0	$2.53\times 10^{-5}$	0
$k_{1,1,2}^{\left(p\right)}$	0	0	0
$k_{2,1,1}^{\left(p\right)}$	0	0	0
$k_{2,1,2}^{\left(p\right)}$	0	0	0
$k_{3,1,1}^{\left(p\right)}$	9.66 × 10${}^{-3}$	8.08 × 10${}^{-3}$	9.45 × 10${}^{-3}$
$k_{3,1,2}^{\left(p\right)}$	0	0	0
$k^{(}{p)}_{1,2,1}$	0	0	0
$k_{1,2,2}^{\left(p\right)}$	0	0	0
$k_{2,2,1}^{\left(p\right)}$	0	0	0
$k_{2,2,2}^{\left(p\right)}$	0	0	0
$k_{3,2,1}^{\left(p\right)}$	0	0	0
$k_{3,2,2}^{\left(p\right)}$	0	0	0

## Data Availability

Data sharing not applicable.
